# Resources needed for US CDC’s support to the response to post-epidemic clusters of Ebola in West Africa, 2016

**DOI:** 10.1186/s40249-018-0484-6

**Published:** 2018-10-12

**Authors:** Cristina Carias, Bishwa B. Adhikari, Fatima Ravat, Martin I. Meltzer, Barbara J. Marston

**Affiliations:** 1 National Center for Emerging and Infectious Zoonotic Diseases, Centers for Disease Control and Prevention, 1600 Clifton Road, MS C18, Atlanta, GA 30329-4027 USA; 2 Center for Global Health, Centers for Disease Control and Prevention, Atlanta, USA

**Keywords:** Ebola cluster, Cost, Emergency response, Post-epidemic

## Abstract

**Background:**

West African countries Liberia, Sierra Leone, and Guinea experienced the largest and longest epidemic of Ebola virus disease from 2014 to 2016; after the epidemic was declared to be over, Liberia, Guinea, and Sierra Leone still experienced Ebola cases/clusters. The United States Centers for Disease Control and Prevention (US CDC) participated in the response efforts to the latter Ebola clusters, by assisting with case investigation, contact identification, and monitoring. This study aims to estimate the cost to the US CDC of responding to three different Ebola clusters after the end of the Ebola epidemic in 2015: i) Sierra Leone, Tonkolili (Jan 2016, 2 Ebola cases, 5 affected regions); ii) Guinea, Nzerekore (Mar-May 2016, 10 Ebola cases, 2 affected regions); iii) Liberia, Somali Drive (Mar 2016, 3 Ebola cases, 1 affected region).

**Main text:**

After interviewing team members that had participated in the response, we estimated total costs (expressed in 2016 US Dollars [USD]), where total costs correspond to travel costs, deployed personnel costs, costs to prepare for deployment, procurement and interagency collaboration costs, among others. We also estimated cost per cluster case (corresponding to the total costs divided by the total number of cluster cases); and cost per case-affected-region (equal to the total costs divided by the product of the number of cases times the number of regions affected). We found that the response cost varied sixteenfold between USD 113 166 in Liberia and USD 1 764 271 in Guinea, where the main cost drivers were travel and personnel costs. The cost per cluster case varied tenfold between 37 722 in Liberia (three cases) and USD 347 226 in Sierra Leone, and the cost per case-affected-region varied threefold between USD 37 722 in Liberia and USD 88 214 in Guinea.

**Conclusions:**

Costs vary with the characteristics of each cluster, with those spanning more regions and cases requiring more resources for case investigation and contact identification and monitoring. These data will assist policy makers plan for similar post-epidemic responses.

**Electronic supplementary material:**

The online version of this article (10.1186/s40249-018-0484-6) contains supplementary material, which is available to authorized users.

## Multilingual abstracts

Please see Additional file 1 for translations of the abstract into the five official working languages of the United Nations.

## Background

From 2014 to 2016, West Africa experienced the largest and longest epidemic of Ebola virus disease (EVD). Over 28 500 cases and 11 300 deaths were reported to the World Health Organization (WHO) from Guinea, Sierra Leone and Liberia [1]. After the WHO declarations of the end of continuous transmission each country experienced additional EVD cases/clusters involving up to 13 infected individuals [2]. Such clusters represented a threat of wider dissemination and therefore a threat for global health security. Thus, public health officials in each country mounted responses aided in part by the United States Centers for Disease Control and Prevention (US CDC), where the CDC’s main inputs related to case investigation and contact identification and monitoring.

To aid planning for future such operations involving the CDC or other international organizations, we analysed costs, from the perspective of the CDC, of emergency responses to three clusters: i) Sierra Leone, Tonkolili (Jan 2016; two Ebola cases; five affected regions); [3] ii) Guinea, Nzérékoré (Mar–May, 2016; ten Ebola cases; two affected regions); [4] iii) Liberia, Somali Drive (Mar, 2016; three Ebola cases; one affected region) [5]. Whereas these clusters and the respective public health responses have previously been reported [6, 7], we quantify here the resources used in these responses.

## Methods

We assessed resource usage during the responses by consulting the teams involved in the response, since specific costs are managed by different centres inside CDC. Such costs are limited to the time scope of the cluster and/or personnel deployment. We consulted in-country CDC team members that were or had been stationed in West Africa and had been in charge of the cluster response. We also consulted personnel located in the Emergency Operations Center in the CDC headquarters in Atlanta responsible for setting up travel, and providing logistical materials for the deployed personnel. We thus interviewed 21 Emergency Operations Center and in-country CDC team members to obtain data on the three emergency responses under study. Since the study was performed from the perspective of the CDC, we did not include resources expended by the Ministries of Health in the affected countries or by other supporting partners.

We included travel costs; deployed personnel time (in hours and wages); funds necessary for collaboration with other agencies; costs needed to for procured materials (such as Rapid Diagnostic Tests), if required; cost of local hires in the region where the response took place; cost of vaccination and medical clearance costs required to personnel preparing to be deployed; transport and other logistical costs such as fuel and security, if required; communication equipment/service for deployed personnel; planning and scheduling travel costs (“backstage costs”); and costs required to reimburse partners for support given for the cluster in terms of personnel and resources for emergency evacuation procedures, if required. When addressing personnel compensation, we considered personnel deployed from the CDC’s Atlanta headquarters, personnel from other agencies assisting in the response via an interagency agreement, and CDC teams aggregated from already stationed and CDC-contracted personnel in the country of the response (i.e., CDC in-country teams). We did not include the costs of personnel stationed in Atlanta that did not travel or was not involved in travel preparation.

We calculated total costs. Given wide variability in total costs, we also estimated cost per cluster case, equal to the total costs divided by the total number of cluster cases; and cost per case-affected-region, which is equal to the total costs divided by the product of the number of cases times the number of regions affected. That is, if there are 10 cases spread over two regions, one would divide total costs by 20. To allow for comparison among countries, we expressed costs in US Dollars (USD) in 2016 prices (USD 2016).

## Results

**Fig.1:**
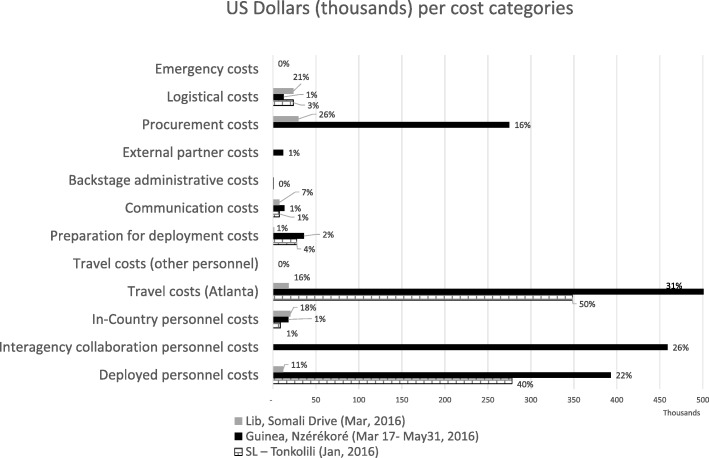
Bar chart representing the magnitude of different cost categories, (in 2016 USD, and as precentage of total costs) for each of the post-epidemic Ebola clusters analysed. Legend: The figure represents the magnitudes of different cost categories. The vertical axis includes the names of all cost categories, while the horizontal axis represents the resources used per category (in thousands of dollars). The different bars correspond to a different post-epidemic Ebola cluster (black: Guinea, Nzérékoré; grey: Liberia, Somali Drive; dotted: Sierra Leone, Tonkolili). Note that the black bar for travel costs for Guinea was truncated (the absolute value was USD 544 550). USD: United States dollars

The cost of responding to an Ebola cluster varied widely between responses, ranging from USD 113 thousand in Liberia, to USD 1 800 thousand in Guinea (Table 1; Fig. 1). In Sierra Leone, response costs were USD 694 452 (Table 1). Regarding deployed-person hours, they varied from 250 h in Liberia to over 5 thousand deployed-person hours for the responses to clusters in Sierra Leone and Guinea; with only Guinea requiring additional personnel from other agencies. In addition, Liberia required 2 040 h from CDC in-country teams, with Guinea and Sierra Leone requiring 5 213 and 1 989 person-hours, respectively.

**Table 1:** Description of cost categories, estimation method, and estimated resource usage (in 2016 USD and person-hours) for each of the post-epidemic Ebola clusters

Cost category	Description	Estimation method	Sierra Leone, Tonkolili (January, 2016) (2 cases; 5 affected regions)^a^ USD (person-hours)	Guinea, Nzérékoré (March 17–May 31, 2016) (10 cases; 2 affected regions)^a^ USD (person-hours)	Liberia, Somali Drive (March, 2016) (3 cases; 1 affected region)^a^ USD (person-hours)
Travel costs for Atlanta personnel	Round-trip travel costs to the West African countries, and per diem (international)	Travel costs were valued at USD 6967 for roundtrip flight, and a per diem of USD 360 that includes lodging and food costs that was multiplied by the duration (in days) of the deployment.	348 181	544 550	18 469
Deployed personnel costs	Opportunity costs of the Atlanta personnel that needs to deployed to West Africa (average hourly personnel salary level GS 13, step 5 assumed)^b^	Team members were asked to estimate personnel hours to assist in the response. Personnel hours were valued at GS13, step 5. Embassy head tax was added for Guinea.	278 110 (5 800h)	392 910 (7 800h)	11 988 (250 h)
Interagency collaboration personnel costs	Opportunity costs of personnel reimbursed by CDC and deployed from other agencies to support the response	Cost was extracted from interagency regulations.	–	459 000 (19 people)	–
Procurement	Cost of rapid diagnostic tests, if deployed	Costs were submitted by team members involved in the response.	–	275 000^c^	29 700^d^
In-country personnel costs	Costs of personnel from West Africa hired for the response (locally employed staff and others)	Team members were asked to estimate local personnel hours. Personnel hours were valued at local contracted rates.	8 611 (1 989 h)	18 026 (5 213 h)	20 345(2 040 h)
Preparation for deployment cost	Costs of vaccination and the medication bag assigned to personnel deployed, medical clearance costs	Cost was extracted from existing agency regulations, and includes Ebola Deployment Medical Clearance and Travel kit.	27 683	35 988	1384
Logistical costs	Costs of fuel and security, if needed	Costs were submitted by team members involved in the response.	23 754	12 500	23 750
Communication costs	Costs of equipment (including laptop, smart phone, GPS device etc.) needed by deployed personnel to communicate from West Africa	Team operatives were asked to provide information on communication materials used; such costs were estimated by subject matter experts.	7500	13 500	7500
Backstage costs	Costs of scheduling and administering travel. Assumed to be 3 h per deployed CDC person	The estimated time to schedule travel was 1 h. The time was multiplied by the number of deployed personnel, and valued at the hourly compensation of backstage personnel (GS10, S5) ^b^.	612	796	31
Partner costs	Resources necessary for CDC partners	Costs required by CDC partners, beyond what was contemplated in the original interagency agreement.	–	12 000	–
Total costs^e^			694 452	1 764 271	113 166

Costs in 2016 values

*CDC* Centers for Disease Control and Prevention, *USD* United States dollars, *GPS* Global positioning system, *RDT* Rapid diagnosis test, *GS/S* General [Government Pay] Schedule/ Step

^a^ For further detail concerning the clusters, please consult references [3–8]

^b^ Government schedule pay tables taken from Office of Personnel Management at https://www.opm.gov/policy-data-oversight/pay-leave/salaries-wages/2017/general-schedule/ (assessed 16 Oct 2017)

^c^ Corresponds to 5000 rapid diagnosis tests

^d^Corresponds to 540 rapid diagnosis tests

^e^ While the authors also enquired about resource use related to required emergency evacuations of personnel (up to USD 250 000), there were none to report for these responses. Also, Foreign Affairs Counter Threat training and clearance costs from Department of State, and World Health Organization were not included – it was assumed that the personnel to be deployed in an emergency response would be experienced and thus would not require such one-time costs at the time of the response

Across all clusters, the biggest drivers of response costs were travel costs (16% in Liberia, 31% in Guinea, and 50% in Sierra Leone) and personnel costs (11% in Liberia, 22% in Guinea, up to 40% in Sierra Leone). These costs are related to the need to deploy personnel expert in epidemiology to perform case investigation, contact identification and monitoring, which were CDC’s main contributions to response (Table 1). Travel costs increase proportionally with the number of responders deployed from Atlanta, and were related to the high costs of air travel and lodging. Deployed personnel costs varied between USD 11 988 in Liberia (three cases, one affected region) and USD 392 910 in Guinea (ten cases, two affected regions). In Sierra Leone (two cases, five affected regions), personnel costs were USD 278 110. Since personnel costs are related to the need for contact tracing, they increase with the complexity of the cluster, that is, with the number of cluster cases and/or regions affected by the cluster.

Other significant costs included are interagency collaboration costs (USD 459 000 in Guinea, or 26% of total costs in this country), which are specific to the particularities of each cluster. The need for these resources increases very rapidly with the number of cases and geographic spread of the cluster. For the responses studied, the complexity of the cluster in Guinea (which included ten cases over two different regions) demanded an additional number of resources to do contact tracing and epidemiologic investigations.

Additionally, the resources required for some responses included procurement costs for rapid diagnostics tests (RDTs) (between USD 29 700 in Liberia, 26% of total costs; and USD 275 000 in Guinea, or 16% of total costs; none in Sierra Leone). The need for RDTs is judged on a case-by-case basis; whether more RDTs are required is a function of the numbers of possibly exposed persons and suspected cases as well as the diagnostic testing algorithm adopted.

In what concerns the resources necessary to prepare for deployment (cost of vaccination, medical clearance), they varied from USD 1384 in Liberia (1% of total costs) to USD 35 988 in Guinea (2% of total costs); with costs in Sierra Leone (USD 27 683; 4% of total costs) also high given the number of regions affected by the cluster and the consequent need to deploy more personnel. Finally, it was also necessary to take into account logistical costs (fuel and security) which varied from almost USD 24 000 in Liberia and Sierra Leone to USD 12 500 in Guinea (these costs correspond to 21% of total costs in Liberia; and less than 3% of total costs in Sierra Leone and Guinea). Such costs depend on the geographic characteristics of the region where the cluster takes place (whether cases are spread over a wide area or not), and on the need or not to hire an extra security detail. Thus, they depend more on the context where the cluster unfolds then on the characteristics of the cluster itself.

The cost per cluster case varied almost tenfold between 37 722 in Liberia (three cases) and USD 347 226 in Sierra Leone (two cases); in Guinea, cost per case was USD 176 427. The cost per case and affected region varied almost threefold between USD 37 722 in Liberia (three cases, one affected region; three cases-affected-regions) and USD 88 214 in Guinea (ten cases, two affected regions; 20 cases-affected-regions); it was USD 69 445 in Sierra Leone (two cases, five affected regions; ten cases-affected regions).

## Discussion

For the clusters studied, the cost per case-affected-region varied almost threefold between USD 37 722 in Liberia and USD 88 214 in Guinea. Total costs varied sixteenfold between USD 113 166 in Liberia and USD 1 764 271 in Guinea. This study illustrates the broad range of resources needed for responding to an international emergency response. The large differences in estimates of cluster costs seem to be due to varied response activity, which depends on the particular characteristics of the cluster (number of cases and number of affected regions). The need for resources seems to increase with both the number of cases and with the geographical dispersion of the cases, with a higher than linear increase on costs per case-affected region.

Note, however, that we did not include costs of personnel that did not travel or were not involved in travel preparation. We also did not include costs from other partners, such as other international organizations and Ministries of Health. That is, the costs presented in this manuscript represent the costs to respond to Ebola clusters, from the perspective of the CDC, and are thus limited to a portion of total response costs. To calculate total costs, the resources used by different partners would also have to be taken into account. Countries that increase their capacity to detect-and-respond to clusters may also experience similar types of response costs, scaled to the size of their Ministries of Health/response teams and the specific requirements for the teams (namely, travel and security requirements). In addition, costs were estimated via interviews; given that we were unable to consult logistical records such as receipts, there may be cost under-reporting. We have tried, however, to collect data for different clusters in order to be able to appreciate the variation of response costs with cluster characteristics (number of cases and regions affected).

## Conclusions

This study is a first step towards quantifying resource usage during the response to an emergent disease outbreak. We found that total response costs varied sixteenfold for the studied outbreaks; they varied threefold by case and region affected. This suggests that the resources required to respond to clusters vary crucially with the characteristics of each outbreak, namely the regions affected and the total number of cases. As a result, a comprehensive assessment of the characteristics of the cluster and regions affected is necessary to gauge the total means necessary to the response. This information is useful for policy makers considering the resources required to mount emergency responses and thereby enhance global health security.

## Additional file

Additional file 1: Multilingual abstracts in the five official working languages of the United Nations. (PDF 192 kb)

## Abbreviations

CDC: Centers for Disease Control and Prevention
EVD: Ebola viral disease
US: United States
USD: United States dollars
WHO: World Health Organization
