# Supplementary material for: Resources needed for US CDC’s support to the response to post-epidemic clusters of Ebola in West Africa, 2016
Source: Infect Dis Poverty. 2018 Oct 12;7:113. doi: 10.1186/s40249-018-0484-6 (PMC6206714; doi:10.1186/s40249-018-0484-6)
Supplement: Supplementary file 1 — Multilingual abstracts in the five official working languages of the United Nations. (PDF 192 kb) [file 40249_2018_484_MOESM1_ESM.pdf]

Translation of the abstract into the five official working languages of the United Nations

الموارد اللازمة لدعم مركز مكافحة الأمراض والوقاية منها (التابع للولايات المتحدة الأمريكية) للاستجابة لمجموعات من حالات الاصابة بـ الإيبولا اللاحقة للوباء في غرب أفريقيا - 2016

كريستينا كارياس، بيشوا ب. أديكاري، فاطمة رافات، باربرا مارستون
Cristina Carias, Bishwa B. Adhikari, Fatima Ravat and Barbara Marston

ملخص

خلفية: شهد غرب أفريقيا أكبر وأطول وباء فيروس إيبولا في الفترة من 2014 حتى 2016. ولقد شاركت مراكز مكافحة الأمراض والوقاية منها (التابعة للولايات المتحدة الأمريكية) في تقديم المساعدة التي تتعلق أساسا بفحص الحالات، وتحديد الاتصال والرصد. تهدف هذه الدراسة إلى تقدير تكلفة استجابة مركز مكافحة الأمراض والوقاية منها (التابع للولايات المتحدة الأمريكية) لثلاث مجموعات مختلفة من حالات الاصابة بـ الايبولا بعد نهاية الوباء سنة 2015.

النص الرئيسي: بعد إجراء مقابلات مع أعضاء الفريق الذي شارك في الاستجابة، قدرنا التكاليف الإجمالية (بالدولار الأمريكي 2016). التكاليف الإجمالية تعادل تكاليف السفر، وتكاليف الأفراد الذين تم نشرهم، وتكاليف التحضير لنشرهم، والمشتريات، وتكاليف التعاون بين الوكالات، من بين أمور أخرى. كما قدرنا التكلفة لكل مجموعة إصابة (تساوي إجمالي التكاليف مقسوما على إجمالي عدد المجموعات)؛ والتكلفة لكل منطقة متأثرة بالحالة (تساوي إجمالي التكاليف مقسوما على حاصل ضرب عدد الحالات بعدد المناطق المتأثرة). ووجدنا أن تكلفة الاستجابة تكاد تصل إلى 20 ضعف فلقد تراوحت بين 105,786 دولاراً أمريكيا في ليبيريا و 1,811,818 دولاراً أمريكيا في غينيا، حيث كان في طليعة التكاليف الرئيسية تكاليف السفر والأفراد. التكلفة لكل مجموعة حالات تراوحت بين 35,262 دولارا أمريكيا في ليبيريا (ثلاث حالات) و 369,290 دولارا أمريكيا في سيراليون (أي بزيادة 10 أضعاف عن مثيلتها في ليبيريا)، والتكلفة لكل منطقة متأثرة بالحالة تراوحت بين 35,262 دولارا أمريكيا في ليبيريا و 90,591 دولارا امريكيا في غينيا (أي بزيادة 3 أضعاف عن مثيلتها في ليبيريا).

الاستنتاجات: التكلفة تختلف تبعاً لخصائص كل مجموعة: تلك التي تغطي المزيد من المناطق والحالات تتطلب المزيد من الموارد لفحص الحالات وتحديد الاتصال والرصد. ستساعد هذه البيانات صناع السياسات على التخطيط لاستجابات مماثلة لما بعد الوباء.

Translated from English version into Arabic by Ammar Alhosain and Ali Mohamed Magdy, through

TRANSLATORS
WITHOUT BORDERS

美国 CDC 援助响应西非埃博拉疫情后期的聚集病例的成本评估

Cristina Carias, Bishwa B. Adhikari, Fatima Ravat and Barbara Marston

摘要

引言：2014 年至 2016 年间，西非地区经历了规模最大、时间最长的埃博拉病毒疫情。美国疾病预防控制中心(US CDC)参与的援助主要涉及病例调查、接触识别和监测。本研究旨在估算埃博拉疫情结束后，US CDC 应对三种不同埃博拉集群的成本。

正文：在采访参与响应的团队成员后，我们估算了总成本(以 2016 年美元表示)，包括差旅成本、部署人员成本、部署准备成本、采购和跨机构协作成本等。我们还估算了每个聚类病例的成本(总成本除以聚类案例总数)；以及每个受影响区域的成本(总成本除以病例数乘以受影响区域数)。我们发现，在利比里亚和几内亚的响应成本相差 20 倍，分别为 105 786 美元和 1 811 818 美元，主要用于差旅和人员花费。利比里亚和塞拉利昂每组病例的响应成本相差 10 倍，分别为 35 262 美元(3 例)和 369 290 美元。利比里亚和几内亚的每个病例影响地区的响应成本相差三倍，分别为 35 262 美元和 90 591 美元。

结论：响应成本因每个集群的特点而存在差异，这些集群跨越了更多的区域且需要更多资源进行病例调查和接触识别和监测。这些数据将有助于决策者对类似的疫情规划应对措施。

Translated from English version into Chinese by Xin-Yu Feng, edited by Jin Chen

CHINESE CENTER FOR DISEASE CONTROL AND PREVENTION
NATIONAL INSTITUTE OF PARASITIC DISEASES

Ressources nécessaires à l’aide apportée par les US CDC dans le cadre de l’intervention au niveau des grappes post-épidémiques du virus Ébola en Afrique de l’Ouest, 2016

Cristina Carias, Bishwa B. Adhikari, Fatima Ravat et Barbara Marston

Résumé
Contexte : L’Afrique de l’Ouest a connu la plus grande et la plus longue épidémie de fièvre Ébola entre 2014 et 2016. Les Centres américains pour le contrôle et la prévention des maladies (US CDC) ont apporté leur aide principalement dans les activités d’enquête de cas, d’identification de contact et de surveillance. Cette étude a pour but d’estimer le coût de l’intervention des US CDC au niveau de trois différentes grappes d’Ébola après la fin de l’épidémie d’Ébola en 2015.
Texte principal : Après avoir interrogé des membres d’équipe ayant participé à l’intervention, nous avons estimé les coûts totaux (exprimés en dollars américains [USD], 2016) correspondant, entre autres, aux frais de déplacement, aux frais de personnel déployé, aux frais de préparation pour le déploiement, aux frais d’approvisionnement et de collaboration interagences. Nous avons également estimé le coût par cas de grappe (correspondant aux coûts totaux divisés par le nombre total de cas de grappes) et le coût par région touchée (correspondant aux coûts totaux divisés par le produit du nombre de cas multiplié par le nombre de régions touchées). Nos résultats ont montré que le coût de l’intervention était multiplié par vingt de 105 786 USD au Libéria à 1 811 818 USD en Guinée, où les principaux inducteurs de coût étaient les frais de déplacement et de personnel. Le coût par cas de grappe était multiplié par dix de 35 262 USD au Libéria (trois cas) à 369 290 USD en Sierra Leone et le coût par région touchée était multiplié par trois de 35 262 USD au Libéria à 90 591 USD en Guinée.
Conclusions : Le coût varie en fonction des caractéristiques de chaque grappe, certaines grappes couvrant plus de régions et de cas et nécessitant plus de ressources pour les enquêtes de cas, l’identification de contact et la surveillance. Ces données sont destinées à faciliter la planification par les responsables politiques d’interventions post-épidémiques similaires.

Translated from English version into French by Maëva P and Elisa Griffin, through

TRANSLATORS
WITHOUT BORDERS

Потребность в ресурсах для обеспечения ЦКП США в целях реагирования на пост-эпидемические кластеры Эболы в Западной Африке в 2016 г.

Кристина Кариас, Бишва Б. Адхикари, Фатима Рават и Барбара Марстон

Аннотация
Предпосылки: С 2014 по 2016 г. Западная Африка пережила самую большую и долгую эпидемию вирусного заболевания Эбола. Центр по контролю и предотвращению заболеваемости США (ЦКП США) участвовал в оказании помощи в основном при расследовании случаев заболевания, идентификации контактов и мониторинге. Настоящее исследование направлено на оценку затрат ЦКП США при реагировании на три различных кластера Эболы после прекращения эпидемии Эболы в 2015 г.
Основной текст: После собеседования с участвовавшими в опросе членами команды мы сделали оценку общих затрат (выраженных в долларах США по курсу 2016 г.), в которых общие затраты, помимо прочих, сопоставляются с затратами на поездки, размещение персонала, подготовку размещения, снабжение и сотрудничество между агентствами. Кроме того, нами оценены затраты на один случай кластера (соответствующие сумме общих затрат, разделенной на общее число случаев кластеров) и затраты на один регион, затронутый кластером (равные сумме общих затрат, разделенной на произведение числа случаев и числа затронутых регионов). Нами обнаружено, что затраты на реагирование различались в двадцать раз от 105 786 долл. США в Либерии до 1 811 818 долл. США в Гвинее, где основными факторами повышения затрат были расходы на поездки и персонал. Затраты на один случай кластера варьировались от 35 262 долл. США в Либерии (три случая) до 369 290

долл. США в Сьерра-Леоне, а затраты на затронутый случаем регион различались троекратно от 35 262 долл. США в Либерии до 90 591 долл. США в Гвинее.
Выводы: Затраты зависят от характеристик каждого кластера; при этом кластеры, охватывающие больше регионов и случаев заболевания, требуют больше ресурсов для расследования этих случаев, идентификации контактов и мониторинга. Эти данные должны помочь принимающим решение лицам при планировании подобных пост-эпидемических реагирований.

Translated from English version into Russian by Alexander Somin and Natalia Potashnik, through

TRANSLATORS
WITHOUT BORDERS

Recursos necesarios para el apoyo de los CDC de EE.UU. a la respuesta a los conglomerados posepidémicos del ébola en África Occidental, 2016

Cristina Carias, Bishwa B. Adhikari, Fatima Ravat y Barbara Marston

Resumen
Antecedentes: África Occidental experimentó la epidemia más grande y duradera de la enfermedad del virus del Ébola entre 2014 y 2016. Los Centros para el Control y la Prevención de Enfermedades de los Estados Unidos (US CDC) se unieron a la ayuda relacionada principalmente con la investigación de casos, la identificación de contactos y la supervisión. Este estudio tiene como objetivo estimar el coste que tuvo para los CDC de EE.UU. responder a tres conglomerados diferentes del ébola después del final de la epidemia de ébola en 2015.
Texto principal: después de entrevistar a los miembros del equipo que habían participado en la respuesta, estimamos los costes totales (expresados en dólares estadounidenses [USD] de 2016), donde los costes totales corresponden a gastos de viaje, gastos de personal desplegado, gastos de preparación para el despliegue, adquisiciones y gastos de colaboración entre agencias, entre otros. También estimamos el coste por caso por conglomerado (correspondiente al coste total dividido por el número total de casos por conglomerado) y el coste por región afectada por caso (igual al coste total dividido por el producto del número de casos multiplicado por el número de regiones afectadas). Encontramos que el coste de la respuesta variaba veinte veces entre 105 786 dólares en Liberia y 1 811 818 dólares en Guinea, donde los principales factores de coste eran los gastos de viaje y de personal. El coste por caso por conglomerado varió diez veces entre 35 262 dólares en Liberia (tres casos) y 369 290 dólares en Sierra Leona, y el coste por región afectada por caso varió tres veces entre 35 262 dólares en Liberia y 90 591 dólares en Guinea.
Conclusiones: el coste varía según las características de cada conglomerado y los que abarcan más regiones y casos requieren más recursos para la investigación de casos y la identificación y supervisión de contactos. Estos datos servirán de ayuda a los responsables de la formulación de políticas para planificar respuestas posepidémicas similares.

Translated from English version into Spanish by Guillermo Pinilla Gallego and Ivana Benzaquen, through

TRANSLATORS
WITHOUT BORDERS
